# Effect of Al Content on Microstructure and Properties of Al_x_Cr_0.2_NbTiV Refractory High-Entropy Alloys

**DOI:** 10.3390/e26060435

**Published:** 2024-05-21

**Authors:** Rongbin Li, Qianqian Li, Zhixi Zhang, Rulin Zhang, Yue Xing, Doudou Han

**Affiliations:** 1 School of Materials, Shanghai Dianji University, Shanghai 201306, China; 2 School of Materials and Chemistry, University of Shanghai for Science and Technology, Shanghai 200093, China; 213353165@st.usst.edu.cn (Q.L.);

**Keywords:** refractory high-entropy alloys, Al content, microstructure, creep

## Abstract

High-temperature creep refers to the slow and continuous plastic deformation of materials under the effects of high temperatures and mechanical stress over extended periods, which can lead to the degradation or even failure of the components’ functionality. Al_x_Cr_0.2_NbTiV (x = 0.2, 0.5, or 0.8) refractory high-entropy alloys were fabricated by arc melting. The effects of Al content on the microstructure of Al_x_Cr_0.2_NbTiV alloys were studied using X-ray diffraction, scanning electron microscopy, and electron backscatter diffraction. The microhardness, compression properties, and nanoindentation creep properties of Al_x_Cr_0.2_NbTiV alloys were also tested. The results show that the Al_x_Cr_0.2_NbTiV series exhibits a BCC single-phase structure. As the Al content increases, the lattice constant of the alloys gradually decreases, and the intensity of the (110) crystal plane diffraction peak increases. Adding aluminum enhances the effect of solution strengthening; however, due to grain coarsening, the microhardness and room temperature compressive strength of the alloy are only slightly improved. Additionally, because the effect of solution strengthening is diminished at high temperatures, the compressive strength of the alloy at 1000 °C is significantly reduced. The creep mechanism of the alloys is predominantly governed by dislocation creep. Moreover, increasing the Al content helps to reduce the sensitivity of the alloy to the loading rate during the creep process. At a loading rate of 2.5 mN/s, the Al_0.8_Cr_0.2_NbTiV alloy exhibits the lowest creep strain rate sensitivity index (m), which is 0.0758.

## 1. Introduction

High-entropy alloys (HEAs) differ markedly from traditional alloys in their compositional complexity. Traditional alloys typically consist of one, or at most two, principal elements. In contrast, HEAs are composed of five or more principal elements mixed in nearly equal atomic ratios. This multi-element mixing strategy significantly enhances the mixing entropy of the alloy, leading to what is referred to as the “high-entropy effect”. This effect can markedly suppress the entropy change associated with the formation of intermetallic compounds, favoring the formation of simple solid solution structures with stronger thermal stability and mechanical properties, such as face-centered cubic (FCC) or body-centered cubic (BCC) crystal structures [1]. These simple solid solutions exhibit many superior properties due to their unique atomic arrangements. For instance, HEAs generally possess higher strength and hardness, excellent wear resistance, corrosion resistance, good oxidation resistance, and radiation damage resistance [2,3].

With the rapid development of high-temperature sectors such as the aerospace and power generation industries, the demand for metallic materials that maintain exceptional mechanical properties under high-temperature conditions has significantly increased. Recent studies have revealed that refractory high-entropy alloys (RHEAs) based on refractory elements such as tungsten (W), molybdenum (Mo), niobium (Nb), and tantalum (Ta) exhibit superior high-temperature strength and good fatigue resistance, surpassing the currently used nickel-based superalloys [4,5]. However, RHEAs, such as the MoNbTaVW alloy [6], typically have high densities and exhibit brittleness at room temperature, which limits their application scope. The addition of aluminum (Al), with its low density and large atomic radius, induces lattice distortions when forming solid solutions with refractory metals, reducing the system’s free energy, thereby enhancing solution strengthening effects. Therefore, Al is often added to RHEAs to reduce alloy density and improve specific strength. Studies have shown that the hard Laves phases induced by Al addition are beneficial to the compressive properties of the Al_x_CrNbTiVZr series alloys [7]. Additionally, the inclusion of Al also contributes to increasing the hardness of the alloys; for example, the Vickers hardness of the Al_x_FeCrVTi_0.25_ alloy displays a trend of initially decreasing and then increasing under the combined effects of solid solution strengthening and second phase strengthening [8].

Structural components used in high-temperature environments often fail due to high-temperature creep. Traditional creep tests, such as uniaxial tensile or compression tests, often require large sample sizes and long testing periods, which has limited the research on the creep properties of RHEAs. To overcome these limitations, the use of nanoindentation techniques for studying creep behavior offers an effective alternative. Nanoindentation is applying a small indentation load on the surface of the material to measure its mechanical properties, allowing for the assessment of creep properties at a very small scale. The advantages of this technique include the small size of samples required and short experimental periods. Existing research indicates that adding Al to CoCrFeNiCu alloys results in a phase transformation from FCC to BCC structures. Although this addition reduces the ductility of the alloy, it effectively enhances its creep resistance at the nanoscale [9]. As the Al content increases, the creep resistance of Al_x_CoCrFeNi (x = 0.3, 0.5, 0.7, 1) alloys shows a trend of initially increasing and then decreasing, but the creep mechanism remains consistent and is characterized by dislocation creep. The alloy exhibits optimal creep resistance when 0.7 mol of Al is added [10]. The refractory high-entropy alloy AlNbTiV, with its single BCC phase and high specific strength [11], emerges as a promising new material for high-temperature applications, yet studies on its creep behavior are limited. This study focuses on a non-equimolar ratio Al_x_Cr_0.2_NbTiV (x = 0.2, 0.5, or 0.8) series of refractory high-entropy alloys. The microstructures of the Al_x_Cr_0.2_NbTiV series of alloys were intensively examined, and their micro- and nanoscale creep behaviors were explored using nanoindentation techniques, providing crucial insights into the creep behavior of refractory high-entropy alloys at micro- and nanoscales.

## 2. Materials and Methods

In this study, alloys of the series Al_x_Cr_0.2_NbTiV (x = 0.2, 0.5, or 0.8 molar ratios) were prepared using a vacuum arc melting furnace in an argon atmosphere, with the process repeated at least four times to ensure chemical uniformity of the ingots. Phase structure analysis was conducted using a Bruker D8 X-ray diffraction (XRD) instrument equipped with a Cu target, covering a scan range of 20° to 90°. Microstructures were observed, and compositions were determined using an S-3400N scanning electron microscope (SEM) (from Hitachi Company, Tokyo, Japan) with a tungsten filament and an attached energy dispersive spectrometer (EDS). Additionally, the grain morphology was analyzed using electron backscatter diffraction (EBSD). In this experiment, the samples were first polished using a 0.4 μm silica suspension and then etched for 15 s with a solution containing 5% hydrofluoric acid and 15% nitric acid to obtain a clear surface. Subsequently, nanoindentation creep tests were conducted using an Anton Paar Step300 nanoindenter (from Anton Paar Company, Graz, Austria) to analyze the micro-deformation mechanisms of the alloy. A standard Berkovich triangular pyramid indenter was used, which applied pressure to the sample at intervals of 20 μm. The peak load was maintained at 30 mN with a holding time of 60 s, and four loading rates were set: 0.1, 0.5, 2, and 2.5 mN/s; the thermal drift was less than 0.3 nm/s. The hardness of the alloy was measured using the HXD-1000 digital microhardness tester (from Shanghai Optical Instrument Factory, Shanghai, China) with a test load of 0.981 N and a dwell time of 10 s. Ten points were tested on each sample, with each point spaced no less than 200 μm apart. To minimize errors, the highest and lowest values were discarded, and the average value of the remaining eight points was taken as the hardness of the alloy. The compressive properties of the alloy at room temperature and 1000 °C were tested using the Gleeble 3180 thermal simulation test machine (from Dynamic Systems Inc., New York, NY, USA) with the strain rate set at 1 × 10^−3^ s^−1^ and a strain of 0.3. Samples were subjected to three sets of parallel experiments at each temperature. Samples for SEM and hardness tests measuring 8 mm × 12 mm × 2 mm, EBSD test samples measuring Φ2 mm × 10 mm, six compression test samples measuring Φ3 mm × 6 mm, and three nanoindentation creep samples measuring 10 mm × 10 mm × 2 mm were prepared using an electrical discharge wire cutting machine.

## 3. Results and Discussion

### 3.1. Effect of Al Content on Phase Structure and Microstructure of High-Entropy Alloys

**Figure 1:**
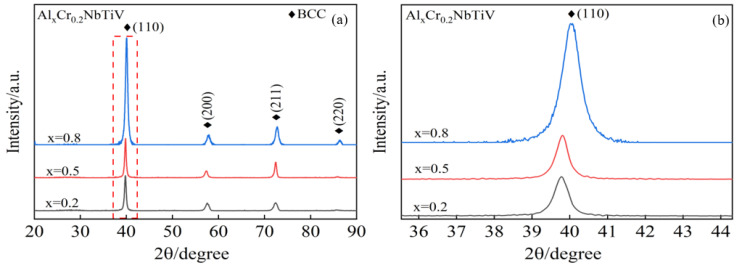
XRD pattern of as-cast Al_x_Cr_0.2_NbTiV HEAs: (**a**) XRD pattern and (**b**) (110) plane pattern.

Figure 1 presents the X-ray diffraction patterns and the diffraction peaks of the (110) planes for the Al_x_Cr_0.2_NbTiV alloys (x = 0.2, 0.5, or 0.8). As shown in Figure 1a, these alloys exhibit a BCC single-phase structure. With the increase in Al content, the intensity of the diffraction peaks at the (110) crystal planes progressively strengthens, and the peak positions shift noticeably towards larger angles, as shown in Figure 1b, with the lattice constant decreasing from 3.198 Å to 3.184 Å. Since Al is a formative element for the BCC structure, increasing the Al content enhances the stability of the BCC phase and hence the strengthening of the (110) diffraction peak [12,13]; moreover, although Al’s atomic radius is larger than that of Cr, it is similar to that of other elements in the alloy. Al enters the alloy via substitutional solid solution, leading to a decrease in the lattice constant, which causes the diffraction peaks to shift towards larger angles [14].

The phase formation rules of high-entropy alloys are primarily associated with mixing enthalpy (Δ*H_mix_*), mixing entropy (Δ*S_mix_*), difference in atomic radii (*δ*), and the valence electron concentration (*VEC*) [15].

Δ*H_mix_* can be used to measure the change in energy of a system under constant temperature and pressure conditions. The calculation formula is as follows:

(1) $${\Delta H}_{mix}=\underset{i=1,i\neq j}{\overset{N}{\sum \Omega_{ij}c_{i}c_{j}}}.$$

In the equation, $\Omega_{ij}=4{\Delta H}_{AB}^{mix}$ represents the interaction parameter for the *i*-th and *j*-th elements in the molten state, and ${\Delta H}_{AB}^{mix}$ is the binary mixing enthalpy between the *i*-th and *j*-th elements.

Δ*S_mix_* is used to measure the randomness of the system at the atomic level. The calculation formula is as follows:

(2) $${\Delta S}_{mix}=-R\underset{i=1}{\overset{N}{\sum c_{i}{lnc}_{i}}}=RLnN.$$

In the equation, *R* represents the gas constant, typically with a value of 8.314 J/(mol·K). *N* is the total number of elements in the alloy system, and *c_i_* is the atomic percentage of the *i*-th element in the alloy.

The *δ* is used to describe the overall effect of atomic size differences in multi-principal-element high-entropy alloys. The calculation formula is as follows:

(3) $$\delta =\sqrt{\underset{i=1}{\overset{N}{\sum c_{i}{\left(1-\frac{r_{i}}{\bar{r}}\right)}^{2}}}}.$$

In the equation, $\bar{r}$ represents the average atomic radius, and *r_i_* is the atomic radius of each element in the alloy.

Previous studies have shown that when Δ*H_mix_* ranges from −15 kJ/mol to 5 kJ/mol, Δ*S_mix_* ranges from 12 J/K·mol to 17.5 J/K·mol, and *δ* is less than 6.2%; high-entropy alloy systems tend to form stable solid solution phases. To predict the specific phase types of alloys (such as BCC, FCC, and HCP), it is necessary to introduce the *VEC*, which predicts the alloy’s microstructure by considering the total number of valence electrons of alloying elements. Its calculation formula is as follows:

(4) $$VEC=\underset{i=1}{\overset{N}{\sum c_{i}{VEC}_{i}}}$$

where *c_i_* represents the atomic percentage of the *i*-th element, and (*VEC*)*_i_* represents the valence electron concentration of the *i*-th element.

When *VEC* < 6.87, the alloy tends to form a BCC structure. A lower *VEC* value reflects a lower density of valence electrons in the alloy, which aids in the formation of the BCC structure with larger interatomic spacing. When 6.87 < *VEC* < 8.0, the alloy exhibits a dual-phase structure of FCC + BCC. When *VEC* > 8.0, the solid solution phase of the FCC structure is relatively stable. The parameters Δ*S_mix_*, Δ*H_mix_*, *δ*, and *VEC* for the cast AlxCr_0.2_NbTiV series of high-entropy alloys are listed in Table 1. The data indicate that increasing the Al content from 0.2 to 0.8 does not alter the phase composition of the alloys, consistent with the results from the XRD diffraction spectra.

**Figure 2:**
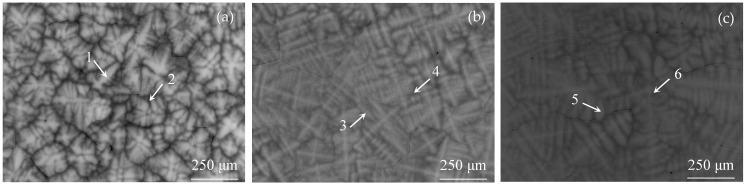
BSE images of as-cast Al_x_Cr_0.2_NbTiV HEAs: (**a**) x = 0.2; (**b**) x = 0.5; and (**c**) x = 0.8.

Figure 2 displays the microstructure of as-cast Al_x_Cr_0.2_NbTiV alloys in backscattered electron (BSE) mode. The alloys exhibit a pronounced dendritic structure with noticeable compositional contrast, indicating the presence of dendritic segregation. According to the EDS compositional analysis results shown in Table 2, the trend of elemental distribution across different regions of the three alloys is similar: the high melting point element Nb preferentially crystallizes in the dendritic (DR) areas, while lower melting point elements such as Ti, Cr, and V enrich in the interdendritic (ID) areas [16]. Moreover, the mixing enthalpy between Al and Nb is −18.2 kJ/mol, where the negative mixing enthalpy strengthens the atomic attraction between them. Consequently, as the content of Al increases, the segregation of Nb in the dendritic areas decreases, and Al aids in distributing Nb more uniformly in the interdendritic regions, thus improving elemental segregation [17,18].

**Figure 3:**
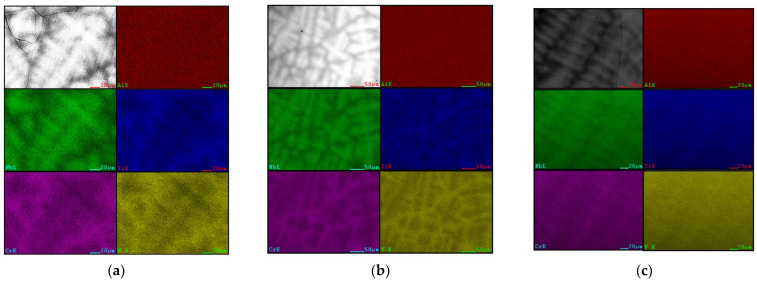
EDS mapping of as-cast Al_x_Cr_0.2_NbTiV HEAs: (**a**) x = 0.2; (**b**) x = 0.5; and (**c**) x = 0.8.

To gain a deeper understanding of the elemental distribution patterns in the Al_x_Cr_0.2_NbTiV alloy and eliminate interference from incidental factors, a mapping of the alloy was conducted using EDS, with the analysis results presented in Figure 3. Through careful observation of the image, significant dendritic segregation phenomena within the alloy were confirmed. Consistent with previous point scan analysis results, the DR regions exhibited enrichment of the high-melting-point Nb element, while the ID regions showed enrichment of other elements with lower melting points.

**Figure 4:**
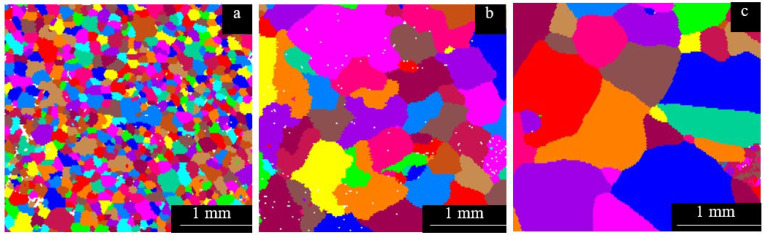
EBSD grain distribution mappings of as-cast Al_x_Cr_0.2_NbTiV HEAs: (**a**) x = 0.2; (**b**) x = 0.5; and (**c**) x = 0.8.

**Figure 5:**
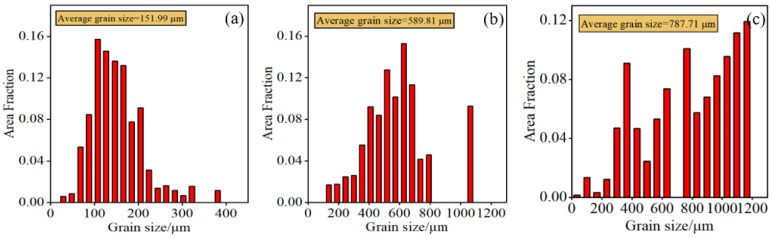
Histogram of grain size of as-cast Al_x_Cr_0.2_NbTiV HEAs: (**a**) x = 0.2; (**b**) x = 0.5; and (**c**) x = 0.8.

Figure 4 presents the EBSD grain distribution maps of the Al_x_Cr_0.2_NbTiV alloys. Due to the rapid cooling of the melt in a water-cooled copper mold, these alloys exhibit irregular equiaxed grain structures. In Figure 5, the average grain sizes of the alloys are 151.99 μm, 589.81 μm, and 787.71 μm. With the increase in Al content, the grain size of the alloy gradually increased. Cr and V, which are elements with smaller atomic radii, can provide nucleation sites, thus refining the grains to some extent [19,20]. However, as the ratios of Al/Cr and Al/V in the Al_x_Cr_0.2_NbTiV series of alloys increase, the grain refining effect gradually diminishes.

### 3.2. Effect of Al Content on Properties of High-Entropy Alloys

**Figure 6:**
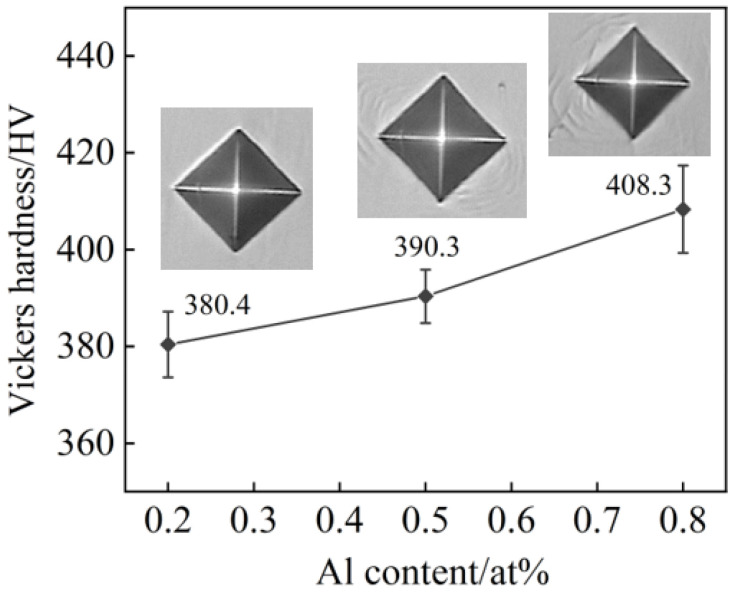
Average Vickers hardness and indentation morphology of as-cast Al_x_Cr_0.2_NbTiV HEAs.

Figure 6 shows the average Vickers hardness values and the corresponding indentation morphologies of alloys with different Al contents.

It can be observed that the Vickers hardness of the alloys slightly increases with the addition of Al. This increase in hardness can be attributed to the intensified lattice distortions caused by the higher Al content, which enhances the solid solution strengthening effect. However, as shown in Figure 4, the grains coarsen with increasing Al content, and according to the Hall–Petch effect, larger grains result in lower hardness [21]. Therefore, despite the increased lattice distortion, the hardness of the alloy only shows a slight improvement. Additionally, as the Al content increases, the brittleness of the alloy also increases [7], as evidenced by the deeper and more numerous cracks around the indentations.

**Figure 7:**
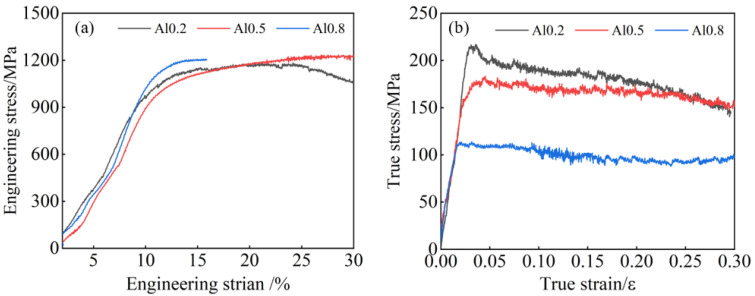
Compressive stress–strain curves of as-cast Al_x_Cr_0.2_NbTiV HEAs: (**a**) room temperature and (**b**) 1000 °C.

As shown in Figure 7, the compression stress–strain curves of the Al_x_Cr_0.2_NbTiV series of alloys indicate that at room temperature, the compressive yield strength does not increase significantly with the addition of Al, following a trend similar to that of hardness. Notably, the alloy with 0.8 Al content fractured during compression, with a fracture strain of 14.5%. At 1000 °C, all alloys exhibited a compressive strain greater than 0.3, but unlike at room temperature, the yield strength gradually decreased with increasing Al content. A data comparison in Table 3 shows that the yield strength of the alloy with 0.2 Al is twice that of the 0.8 Al alloy. Although, generally, an increase in lattice distortion enhances the compressive strength of materials, higher deformation temperatures provide suitable thermal activation conditions, potentially weakening the solid solution strengthening effect [22,23]. Therefore, during compression testing at 1000 °C, increasing Al content paradoxically leads to a reduction in alloy strength.

**Figure 8:**
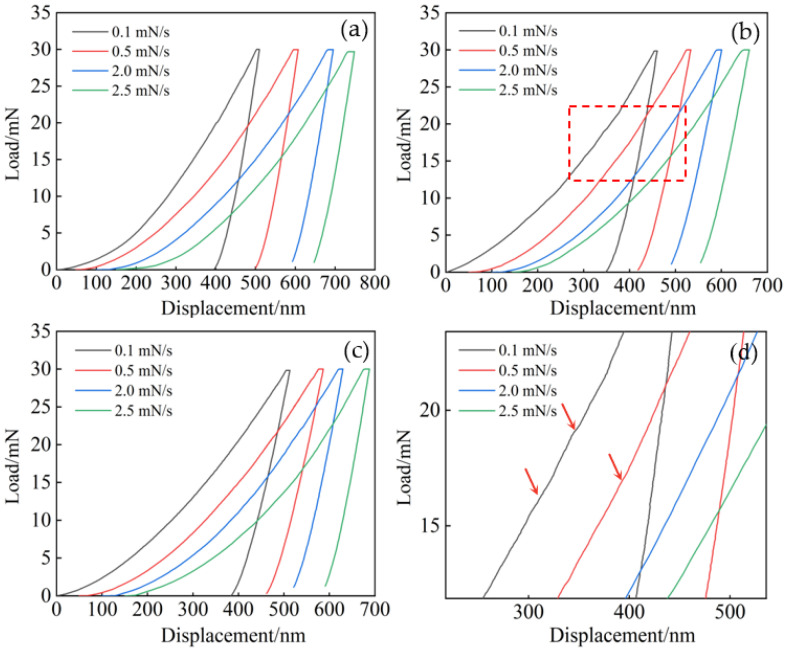
Load–displacement curves of as-cast Al_x_Cr_0.2_NbTiV HEAs: (**a**) x = 0.2; (**b**) x = 0.5; (**c**) x = 0.8; (**d**) partial magnification image of (**b**).

Figure 8a–c displays the load–displacement (P–h) curves obtained from nanoindentation tests of the Al_x_Cr_0.2_NbTiV series of alloys. To facilitate distinction, the curves are horizontally shifted by 50 nm. During the loading phase, the P–h curves at various loading rates are parallel to each other, indicating the reliability of the experimental data. In the hold phase, the load remains constant while the displacement increases over time, suggesting creep occurrence. After unloading, only a minor amount of elastic recovery is observed, indicating that the alloys have undergone permanent deformation. At a loading rate of 0.5 mN/s, the maximum displacements of the alloys with different Al contents are 450 nm, 368 nm, and 411 nm, showing a trend of initially decreasing and then increasing with added Al content. Similar trends are observed at loading rates of 0.1 mN/s, 2.0 mN/s, and 2.5 mN/s. Furthermore, the maximum displacements of all three alloys increase with the increase in loading rate. Due to the strain rate sensitivity of many materials, their hardness decreases with increasing loading rate. At slower rates, plastic deformation mechanisms (such as dislocation movement and interaction) occur over a longer period, resulting in higher resistance to deformation and thus reduced displacement. Conversely, at higher rates, deformation may become more localized, leading to greater displacement and seemingly lower hardness [24].

Figure 8d is a partial magnification image of Figure 8b. In this image, the occurrence of pop-in events can be seen on the P–h curve during the loading phase of the x = 0.5 alloy, indicating the alloy’s response to dislocation nucleation. The sources of nucleation typically involve pre-existing dislocations or vacancies [25,26]. When the loading rate is 0.1 mN/s, the lower loading rate allows sufficient time for dislocations to nucleate and propagate [27], and with a smaller strain gradient, dislocation slip is more likely to occur. This leads to multiple pop-in events on the P–h curve, which appear earlier and are more numerous as indicated by the arrows in Figure 8d.

By fitting the load–displacement curves with an empirical formula (with a confidence level greater than 95.2%) [28], a relationship can be derived between creep displacement and time:

(5) $$H=h_{0}+{a(t_{1}-t_{0})}^{b}+ct_{1}.$$

In the formula, *H* represents the instantaneous indentation depth (nm); *h*_0_ is the indentation depth at the start of creep (nm); *t*_1_ denotes time (s); *t*_0_ is the start time of creep (s); and *a*, *b*, and *c* are the fitting parameters.

**Figure 9:**
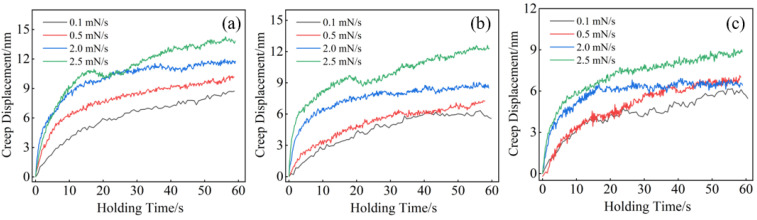
Creep displacement–time curves of as-cast Al_x_Cr_0.2_NbTiV HEAs: (**a**) x = 0.2; (**b**) x = 0.5; and (**c**) x = 0.8.

The creep displacement–time curves during the hold process in Figure 9. As the loading rate increases, the creep displacement also increases, and the creep rate during the transient phase accelerates significantly. Due to the higher loading rate, creep deformation occurs within a short period of time. Therefore, during the loading process, relatively shorter time-dependent relaxation results in more significant creep deformation during the dwell phase. Conversely, at lower loading rates, longer loading times lead to a greater extent of time-dependent relaxation [24,29]. At a loading rate of 2.5 mN/s, the creep depths of the alloys are 13.8 nm, 12.3 nm, and 8.7 nm. These results indicate that with an increase in Al content, the alloy’s creep resistance is enhanced. This improvement is primarily attributed to two factors. First, the alloy is a single-phase BCC material with fewer slip systems and higher stacking fault energy, and the addition of Al enhances the stability of the BCC phase, as evidenced by the intensified diffraction peak of the (110) crystal plane shown in Figure 1b [30]. Second, as the Al content increases, the mixing enthalpy of the alloy becomes more negative (see Table 1), suggesting a higher entropy effect that makes atomic movement more difficult [31], thereby enhancing the alloy’s creep resistance.

Based on the creep displacement–time curves, the equivalent stress and creep rate of the alloy can be calculated as follows [32]:

(6) $$\dot{\varepsilon }=\frac{1}{h}\frac{d_{h}}{d_{t_{1}}}$$

(7) $$\sigma =\frac{P_{\max}}{ch^{2}}.$$

In the formula, *έ* represents the creep rate and *σ* denotes the equivalent stress. *P_max_* is the peak load, while *c* is the geometrical coefficient for the hardness indenter. For a Berkovich indenter, the *c* value is 24.56 [33].

Similar to traditional alloys, the *έ* of high-entropy alloys also follows a power–law relationship with the *σ*. The creep stress exponent *n* is determined using the following Formula (8) [34]:

(8) $$n=\frac{\partial \ln\dot{\varepsilon }}{\partial \ln\sigma }.$$

To further analyze the creep deformation mechanisms of the alloys, the dislocation activation volume during the creep process can be calculated as follows [35]:

(9) $$V^{*}=\frac{\sqrt{3}kTn}{H}.$$

In the formula mentioned, *k* represents the Boltzmann constant, *H* denotes the nanoindentation hardness (GPa), and *T* is the temperature (K).

**Figure 10:**
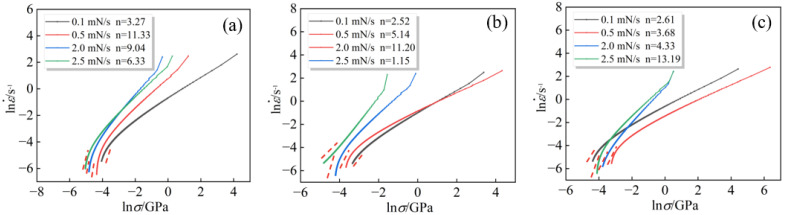
Creep stress exponent fitting curve of as-cast Al_x_Cr_0.2_NbTiV HEAs: (**a**) x = 0.2; (**b**) x = 0.5; and (**c**) x = 0.8.

According to Formula (4), the creep stress exponent curve of the alloy can be fitted, and the value of the creep stress exponent *n* can be derived from the slope of the curve during the steady state, as indicated by the red dotted line in Figure 10a–c. When *n* < 1, it suggests that the creep mechanism of the alloy is diffusion creep; when 1 < *n* < 2, it corresponds to the grain boundary creep mechanism; and when *n* > 3, it indicates the dislocation creep mechanism [36,37]. For the Al_x_Cr_0.2_NbTiV series of alloys, increasing the Al content does not alter the creep mechanism when the loading rate is below 2.5 mN/s. In this case, the creep stress exponent n values are all greater than three, indicating that the creep deformation is primarily accomplished through dislocation slip and climb.

In addition to the creep stress exponent n, the creep strain rate sensitivity m is another crucial metric for assessing the creep resistance of materials; a lower *m* value indicates better creep resistance. There is a specific relationship in which *m* = 1/*n*. It has been found that for the alloy with Al content x = 0.2, the *m* value initially decreases and then increases with increasing loading rate. When the Al content increases to x = 0.5, the *m* value follows a similar trend of initially decreasing and then increasing. Further increasing the Al content to x = 0.8, the *m* value of the alloy decreases with increasing loading rate, reaching 0.0758 at a loading rate of 2.5 mN/s, which is approximately one-third of the *m* value for the CoCrFeCuNi alloy studied by Ma et al. [38]. This indicates that an increase in Al content helps to reduce the sensitivity of the Al_x_Cr_0.2_NbTiV alloys to loading rate during creep.

**Figure 11:**
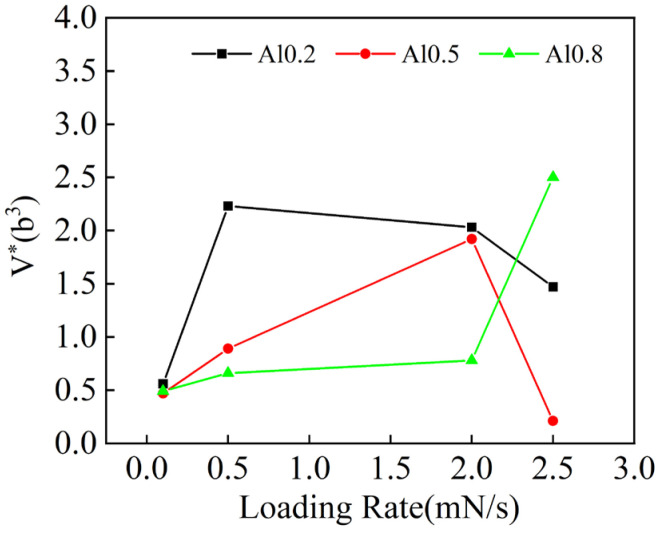
Change curve of dislocation activation volume with loading rate of as-cast Al_x_Cr_0.2_NbTiV HEAs.

When the loading rate increases to 2.5 mN/s, the creep stress exponent n for the alloy with x = 0.5 abruptly shifts to 1.15, as illustrated in Figure 10b. This change indicates that under these conditions, the alloy’s creep mechanism transitions to diffusion creep. At this point, the alloy’s dislocation activation volume V* is 0.21 b^3^ (Figure 11), which is typically associated with the non-uniform nucleation of dislocations, and creep deformation occurs through direct atom–vacancy exchange [39]. These findings suggest that the creep deformation mechanism of the same alloy can vary under different loading rates. At high loading rates, the alloy may exhibit creep behavior distinct from that observed at lower rates.

## 4. Conclusions

1. Al_x_Cr_0.2_NbTiV alloys (x = 0.2, 0.5, or 0.8) all exhibit a BCC single-phase structure with a dendritic microstructure. With increasing Al content, there is a reduction in Nb segregation, coarsening of grains, reduction in lattice parameters, intensification of lattice distortions, and an increase in the intensity of the (110) crystal plane diffraction peaks.
2. Adding aluminum enhances the effect of solution strengthening; however, due to grain coarsening, the microhardness and room temperature compressive strength of the alloy are only slightly improved. Additionally, because the effect of solution strengthening is diminished at high temperatures, the compressive strength of the alloy at 1000 °C is significantly reduced.
3. The creep resistance of the alloys increases with increasing Al content. Under loading rates less than 2.5 mN/s, the creep stress exponent *n* is greater than three, indicating that the coarse-grained RHEAs with a BCC single-phase structure primarily deform through dislocation creep mechanisms at low stress and slow loading rates.
4. Increasing Al content reduces the sensitivity of the alloys to loading rates during creep. Particularly in the Al_0.8_Cr_0.2_NbTiV alloy, as the loading rate increases, the creep strain rate sensitivity m decreases, reaching 0.0758 at a loading rate of 2.5 mN/s.

## Figures and Tables

**Table 1:** Parameters of as-cast Al_x_Cr_0.2_NbTiV HEAs, including Δ*H_mix_*, Δ*S_mix_*, *δ*, and *VEC*.

Alloys	Δ*H_mix_*/kJ·mol^−1^	Δ*S_mix_*/J·K^−1^·mol^−1^	*δ*/%	*VEC*
x = 0.2	−5.96	11.75	5.4	4.674
x = 0.5	−10.82	12.38	5.2	4.513
x = 0.8	−14.21	12.57	5.0	4.400

**Table 2:** EDS analysis results of different regions in the as-cast Al_x_Cr_0.2_NbTiV HEAs.

Alloys	Regions	Al	Cr	Nb	Ti	V
x = 0.2	1 (DR)	5.34	5.03	31.45	29.00	29.17
	2 (ID)	5.84	6.33	25.06	31.99	30.79
x = 0.5	3 (DR)	11.15	5.11	26.63	28.71	28.39
	4 (ID)	11.71	6.22	22.69	30.25	29.13
x = 0.8	5 (DR)	18.34	4.34	25.92	25.75	25.65
	6 (ID)	19.04	5.91	21.76	27.33	25.96

**Table 3:** Compressive mechanical properties of the as-cast Al_x_Cr_0.2_NbTiV HEAs.

Temperature (°C)	Strength (MPa)	x = 0.2	x = 0.5	x = 0.8
Room temperature	Yield strength	978.3	951.3	1040
	Compressive strength	1199.5	1226.6	1209.5
	Fracture strain	>30%	>30%	14.5%
1000	Yield strength	209	160	105
	Compressive strength	215	178	113
	Fracture strain	>30%	>30%	>30%

## Data Availability

Data are contained within the article.
